# AP-1-mediated chromatin looping regulates ZEB2 transcription: new insights into TNFα-induced epithelial–mesenchymal transition in triple-negative breast cancer

**DOI:** 10.18632/oncotarget.3158

**Published:** 2015-01-30

**Authors:** Yichun Qiao, Chiou-Nan Shiue, Jian Zhu, Ting Zhuang, Philip Jonsson, Anthony P.H. Wright, Chunyan Zhao, Karin Dahlman-Wright

**Affiliations:** ^1^ Department of Biosciences and Nutrition, Novum, Karolinska Institutet, Huddinge, Sweden; ^2^ Clinical Research Center (KFC), Department of Laboratory Medicine, Karolinska Institutet, Huddinge, Sweden; ^3^ Department of Pediatrics, Hualien Tzu Chi Hospital, Buddist Tzu Chi Medical Foundation, Hualien, Taiwan; ^4^ Center for Nuclear Receptors and Cell Signaling, Department of Biology and Biochemistry, University of Houston, Houston, USA; ^5^ Science for Life Laboratory, Karolinska Institutet, Solna, Sweden

**Keywords:** AP-1, triple-negative breast cancer, ZEB2, epithelial–mesenchymal transition, TNFα

## Abstract

The molecular determinants of malignant cell behaviour in triple-negative breast cancer (TNBC) are poorly understood. Recent studies have shown that regulators of epithelial-mesenchymal transition (EMT) are potential therapeutic targets for TNBC. In this study, we demonstrate that the inflammatory cytokine TNFα induces EMT in TNBC cells via activation of AP-1 signaling and subsequently induces expression of the EMT regulator ZEB2. We also show that TNFα activates both the PI3K/Akt and MAPK/ERK pathways, which act upstream of AP-1. We further investigated in detail AP-1 regulation of ZEB2 expression. We show that two ZEB2 transcripts derived from distinct promoters are both expressed in breast cancer cell lines and breast tumor samples. Using the chromosome conformation capture assay, we demonstrate that AP-1, when activated by TNFα, binds to a site in promoter 1b of the *ZEB2* gene where it regulates the expression of both promoter 1b and 1a, the latter via mediating long range chromatin interactions. Overall, this work provides a plausible mechanism for inflammation-induced metastatic potential in TNBC, involving a novel regulatory mechanism governing ZEB2 isoform expression.

## INTRODUCTION

Triple-negative breast cancer (TNBC), a tumor type defined by lack of expression of the estrogen, progesterone and HER2 receptors, represents a more aggressive and metastatic form of breast cancer than other types of breast cancer [1]. Furthermore, treatment of patients with TNBC is challenging. Whilst breast tumors that express estrogen receptor (ER) are responsive to ER antagonists and aromatase inhibitors and HER2-positive tumors are effectively treated with HER2-blocking antibodies and/or HER2 kinase inhibitors [2], TNBC lacks a targeted therapy. Understanding the molecular pathways determinant for TNBC behaviour is one strategy for developing more effective treatments for TNBCs.

The transcription factor AP-1 consists of different dimeric combinations of either homodimers of Jun proteins (c-Jun, JunB and JunD) or heterodimers of Jun and Fos proteins (c-Fos, FosB, Fra-1 and Fra-2). AP-1 regulates the expression of AP-1 target genes after binding to 12-*O*-tetradecanoylphorbol-13-acetate (TPA) response elements (TREs) associated with regulated genes [3]. Upon activation by growth factors, hormones or cytokines, AP-1 signaling plays a critical role in numerous cellular processes, including proliferation, differentiation, apoptosis, cell migration, and transformation [4].

TNFα, one of the inflammatory cytokines, signals via TNF receptors, TNFRI and TNFRII. Increasing evidence indicates that a chronic and consistent expression of TNFα in tumors promotes tumor development and progression [5]. Specifically in relation to breast cancer, TNFα was found to be highly expressed in breast tumors as compared with normal tissues [6], and patients with more advanced breast tumor phenotypes were shown to have elevated TNFα serum levels [6, 7]. TNFα promotes breast cancer cell proliferation and migration *in vitro* [8] and contributes to mammary tumorigenesis *in vivo* [9]. In addition, depletion of TNFα expression in a TNBC cell line led to apoptosis and inhibition of cell proliferation, indicating that TNFα plays a fundamental role in the promotion and progression of TNBC [10].

ZEB2 belongs to a small family of transcriptional factors characterized by containing a homeo domain flanked by two separated zinc finger clusters [11]. It is expressed in various types of human tumors, such as breast cancer, gastric cancer, and ovarian cancer [11]. ZEB2 is a potent repressor of E-cadherin through its direct binding to the E-cadherin promoter and a key player in tumor cell invasion and metastasis [12, 13]. Consequently, understanding the structure and the regulation of the *ZEB2* gene is critical.

Gene looping is increasingly recognized to play important regulatory roles in gene expression [14]. The use of recently developed techniques such as chromosome conformation capture (3C) has revealed that higher-order chromatin structure involves long-range loop formation between distant genomic elements [15]. Long-range interactions between promoters and distal elements have been discovered in a wide variety of gene loci, and the formation of looping interactions is significantly correlated with gene expression [16].

Epithelial-to-mesenchymal transition (EMT) is a cellular process critical to normal morphogenesis but also cancer metastasis [17–19]. EMT can be triggered by different signals received from tumor microenvironments, such as TNFα, TGFβ, EGF, WNTs and Notch [18–20]. During the EMT, epithelial cells acquire fibroblast-like properties and show reduced intercellular adhesion and enhanced motility [21]. One of the hallmarks of EMT is loss of expression of the cell-cell junction protein E-cadherin [17–19]. Several transcription factors, including Snail, Slug, ZEB1, Twist, and ZEB2, have been shown to act as master regulators of the EMT program [22–24]. We have recently reported that AP-1 promotes cell invasion through transcriptional upregulation of ZEB2 in TNBC cells [13]. In this study, we further dissect the AP-1–ZEB2 axis in TNFα-induced EMT in TNBC cells.

## RESULTS

### TNFα induces EMT in TNBC cells

**Figure 1:**
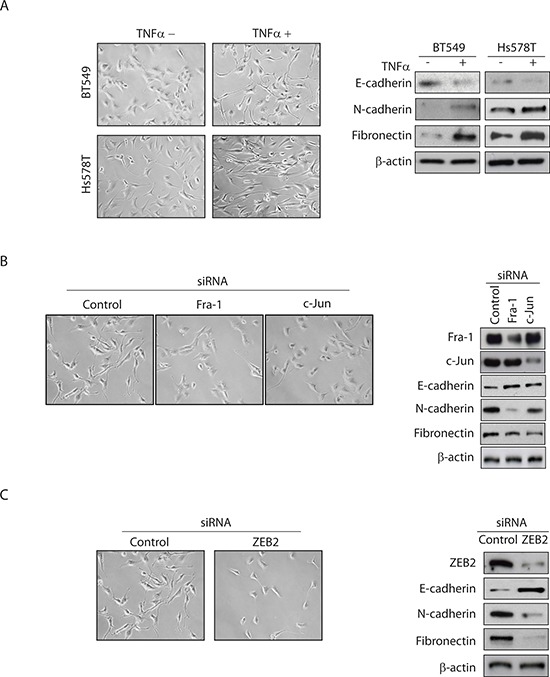
TNFα-mediated EMT in TNBC cells is dependent on AP-1–ZEB2 signaling **(A)** BT549 and Hs578T cells were serum-starved overnight and then treated with or without TNFα (10 ng/ml) for 72 hours. The cells were examined by phase contrast microscopy and Western blots analysis for epithelial (E-cadherin) and mesenchymal (N-cadherin and fibronectin) markers. β-actin was used as a loading control. Representative images from two independent studies. **(B)** BT549 cells transfected with control, Fra-1 or c-Jun siRNA were treated as above. Western blot confirms knockdown of Fra-1 or c-Jun levels. **(C)** BT549 cells transfected with control or ZEB2 siRNA were treated as above. Western blot confirms knockdown of ZEB2 levels. The same image for control siRNA is shown for Figure 1B and Figure 1C because all conditions were assessed in the same experiment.

EMT is characterized by down-regulation of epithelial markers such as E-cadherin and up-regulation of mesenchymal markers such as N-cadherin and fibronectin. Figure 1A shows that the TNBC cell lines BT549 and Hs578T acquired EMT-like morphological features, such as a spindle-shaped appearance, in response to TNFα treatment. In agreement with the change in cellular appearance, TNFα treatment led to significant reduction in E-cadherin protein expression as well as increases in N-cadherin and fibronectin protein expression (Figure 1A), all characteristics of EMT.

### TNFα-mediated EMT in TNBC cells is dependent on AP-1–ZEB2 signaling

We have previously demonstrated that AP-1 signaling, via activation of ZEB2, induces cell invasion of TNBC cells [13]. We have also demonstrated that Fra-1 and c-Jun are highly expressed AP-1 protein family members in these cells [13]. In the following set of experiments, we explored whether the AP-1–ZEB2 axis also mediates TNFα-induced EMT in TNBC cells.

We depleted Fra-1 or c-Jun (Figure 1B) and ZEB2 (Figure 1C) in BT549 cells, and analysed cellular appearance and EMT markers in TNFα-stimulated cells. Whereas control cells underwent marked EMT after 3 days of TNFα treatment, Fra-1- or c-Jun-depleted cells (Figure 1B) and ZEB2-depleted cells (Figure 1C) did not acquire EMT characteristics in the form of spindle-like morphological features. Similar results were obtained with an independent pool of Fra-1, c-Jun or ZEB2 siRNAs (data not shown), suggesting that this effect is specific to Fra-1, c-Jun and ZEB2 silencing. Consistent with the observed effect on morphological features, Fra-1, c-Jun, or ZEB2 knockdown increased the expression level of E-cadherin, and decreased the expression levels of N-cadherin and fibronectin (Figure 1B and 1C). Combined, these data suggest that the AP-1–ZEB2 axis is required for TNFα-mediated EMT in TNBC cells.

### TNFα induces AP-1 activation and ZEB2 expression in TNBC cells

We have previously demonstrated that AP-1 activation downstream of the PI3K/Akt and MAPK/ERK signaling pathways transactivates ZEB2 gene expression [13]. To gain additional insight into the molecular details of the AP-1–ZEB2 axis in TNFα-induced EMT in TNBC cells and in particular, if our previous finding regarding AP-1 activation of ZEB2 can be translated to the effects of TNFα in this system, we assayed AP-1 activation and ZEB2 expression in response to TNFα treatment in TNBC cells.

**Figure 2:**
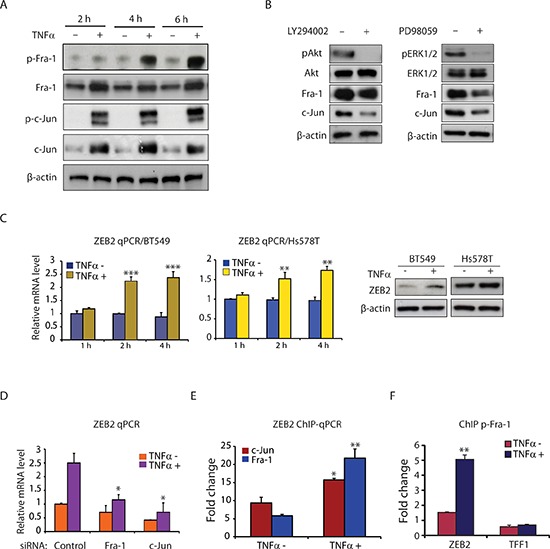
TNFα induces AP-1 activation and ZEB2 expression in TNBC cells **(A)** Western blot analysis of p-Fra-1, Fra-1, p-c-Jun, c-Jun, and β-actin (as a loading control) in BT549 cells treated with or without TNFα (10 ng/ml) for 2, 4 and 6 hours. **(B)** TNFα mediates Fra-1 and c-Jun expression via the PI3K/Akt and MAPK/ERK pathways. Western blot analysis of pAkt, Akt, pERK1/2, ERK1/2, Fra-1, c-Jun and β-actin (as a loading control) in BT549 cells pretreated with LY294002 (25 μM) or PD98059 (25 μM) for 6 hours, followed by stimulation with TNFα (10 ng/ml) for 6 hours. **(C)** TNFα induces ZEB2 expression at both the mRNA and protein levels in BT549 and Hs578T cells. ZEB2 mRNA levels were determined by qPCR after treatment with or without TNFα (10 ng/ml) for the indicated times. Data are shown as means with SD. **p < 0.01, ***p < 0.001 compared with TNFα minus (*n* = 3). The protein levels of ZEB2 after 6 hours of treatment with TNFα were analyzed by Western blot. β-actin was used as a loading control. **(D)** Depletion of Fra-1 or c-Jun markedly impaired the induction of ZEB2 gene expression by TNFα. Values are mean ± SD (*n* = 3). *p < 0.05 compared with control siRNA. **(E)** ChIP-qPCR analysis showing increased binding of Fra-1 and c-Jun to *ZEB2* following TNFα treatment. Columns, mean fold enrichment of Fra-1 or c-Jun relative to IgG; bars, SD (*n* = 3). *p < 0.05, **p < 0.01 compared with TNFα minus. **(F)** ChIP-qPCR analysis showing increased binding of phospho-Fra-1 to *ZEB2* following TNFα treatment. *TFF1* is used as a negative control. ChIP was performed with antibody against phospho-Fra-1. Columns, mean fold enrichment of phospho-Fra-1 relative to IgG; bars, SD (*n* = 3). **p < 0.01 compared with TNFα minus.

As shown in Figure 2A, TNFα treatment markedly up-regulated the levels of phospho-Fra-1, Fra-1, phospho-c-Jun, and c-Jun proteins at 2, 4 and 6 hours. To determine whether the PI3K/Akt and MAPK/ERK signaling pathways mediate TNFα up-regulation of AP-1 levels and activity, we used two pharmacological inhibitors, LY294002 and PD98059, to block the PI3K/Akt and MAPK/ERK pathways, respectively. We found that LY294002 and PD98059 in TNFα-stimulated cells inhibited TNFα-induced expression of Fra-1 and c-Jun proteins (Figure 2B), suggesting that activation of the PI3K/Akt and MAPK/ERK pathways is responsible for TNFα-induced AP-1 protein expression. The effect of inhibitor treatment on these pathways was confirmed by assessing the level of Akt and ERK1/2 phosphorylation (Figure 2B). We next examined ZEB2 expression after TNFα stimulation. Compared to untreated cells, TNFα significantly increased ZEB2 mRNA levels after 2 and 4 hours of treatment in the BT549 and Hs578T cell lines with a subsequent increase in ZEB2 protein levels (Figure 2C). Finally, a role of AP-1 signaling in mediating TNFα-induced up-regulation of ZEB2 was provided by depleting Fra-1 or c-Jun in BT549 cells prior to TNFα stimulation. Depletion of Fra-1 or c-Jun markedly impaired the induction of ZEB2 gene expression by TNFα (Figure 2D). Furthermore, TNFα treatment increased binding of Fra-1 and c-Jun to an AP-1 binding region in an intron of the *ZEB2* gene that we have previously described [13] (Figure 2E). To further determine whether phospho-Fra-1 is binding to DNA, we assessed the ability of phospho-Fra-1 to bind to the *ZEB2* gene. Consistent with the markedly elevated levels of phospho-Fra-1 upon TNFα treatment, phospho-Fra-1 binding was increased by TNFα (Figure 2F). Collectively, these results show that TNFα-induced AP-1 activation, via distinct pathways, is a critical mediator of ZEB2 up-regulation in TNBC cells.

### TNFα regulates ZEB2 via two promoters dependent on AP-1 in TNBC cells

**Figure 3:**
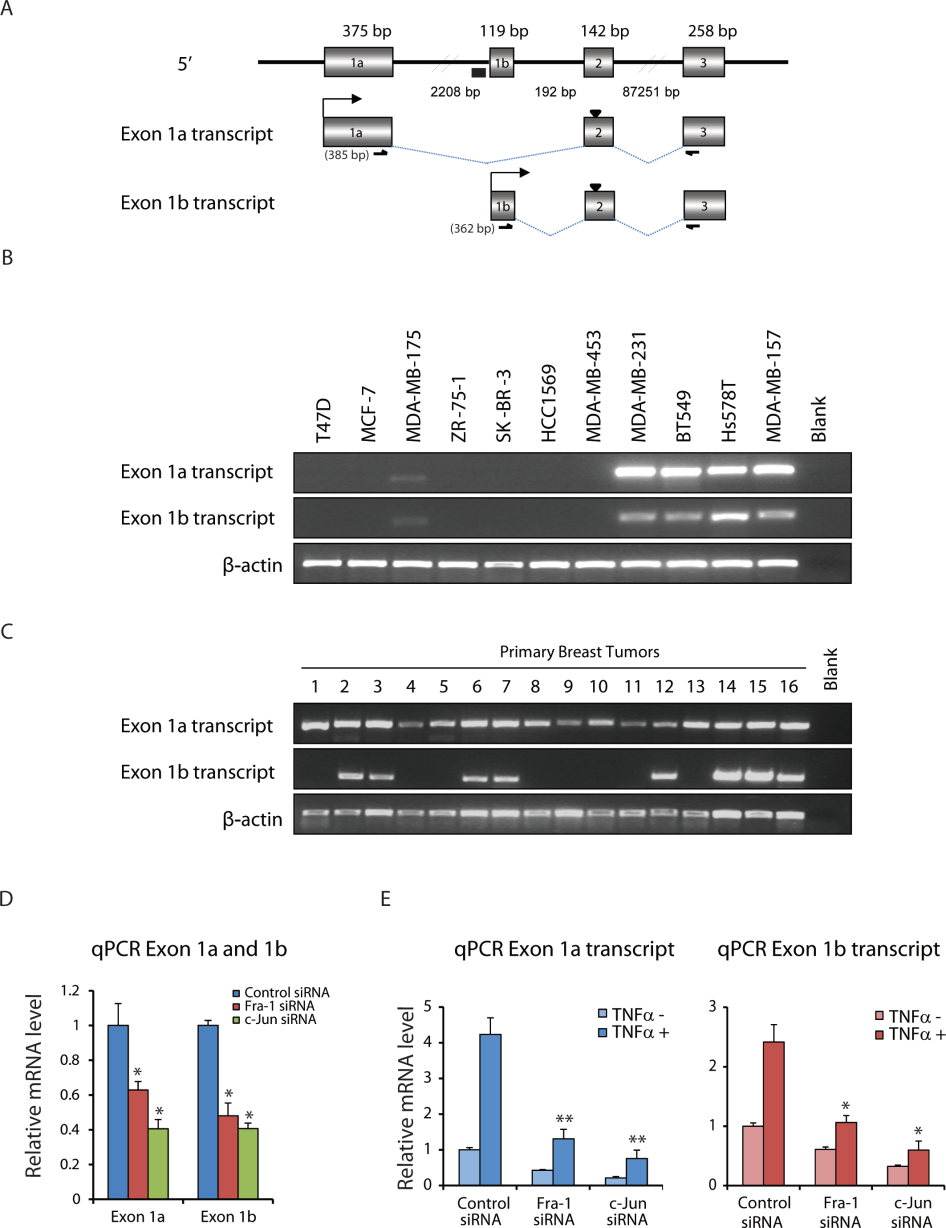
TNFα regulates ZEB2 via two promoters dependent on AP-1 in TNBC cells **(A)** Structure of the 5′ region of the *ZEB2* gene and schematic of mRNA initiated from two distinct first exons, named exon 1a and exon 1b, splicing to downstream exon 2. Origins of sequences: exon 1a was from ENST00000558170, and exon 1b from ENST00000409487. The size of each exon and distances between exons are indicated. ‘▼’ at exon 2 indicates ATG, ‘▄’ indicates AP-1 binding site and bent arrows indicate transcriptional start sites. RT-PCR primer sets to amplify exon 1a and exon 1b transcripts are also indicated. Expected sizes of the products are shown in the parentheses. **(B)** RT-PCR detection of the expression of exon 1a and exon 1b transcripts in RNA isolated from breast cancer cell lines. Expression of β-actin mRNA was used as an internal control. **(C)** RT-PCR detection of the expression of exon 1a and exon 1b transcripts in breast tumor RNA. **(D)** qPCR data showing decreased exon 1a and exon 1b transcript levels in BT549 cells upon Fra-1 or c-Jun depletion. Values are mean ± SD (*n* = 3). *p < 0.05 compared with control siRNA. **(E)** qPCR data showing that depletion of Fra-1 or c-Jun markedly impaired the induction of exon 1a and exon 1b transcript expression by TNFα. Values are mean ± SD (*n* = 3). *p < 0.05, **p < 0.01 compared with control siRNA.

As ZEB2 participates in regulation of EMT, a detailed knowledge of mechanisms for its expression is of fundamental importance. Human ZEB2 mRNAs initiate from two distinct first exons, referred to as exon 1a and exon 1b, respectively, which are located 2.2 kb apart and are spliced to the common exons 2–10 (Figure 3A). We examined the expression of exon 1a and exon 1b transcripts in a panel of breast cancer cell lines and primary human breast tumors. As shown in Figure 3B, among 11 breast cancer cell lines, expression of the two transcripts was detected in 5 cell lines. In primary breast tumors, the exon 1a transcript was detected in all analyzed samples, whilst the exon 1b transcript displayed a more restricted expression (Figure 3C). Together, these results reveal that two alternatively spliced 5′UTR isoforms of the *ZEB2* gene can be expressed in breast cancer cell lines and breast cancer tissues, indicating that the two promoters, driving the respective transcripts, are functional.

Using ChIP-seq, we have previously found that the *ZEB2* gene harbors only one AP-1 binding site that is located in the promoter 1b region (Figure 3A) and based on this, we hypothesized that AP-1 regulation of ZEB2 is restricted to the ZEB2 exon 1b transcript. However, as shown in Figure 3D, expression of transcripts including exon 1a as well as expression of transcripts including exon 1b were decreased significantly after the knockdown of Fra-1 or c-Jun (Figure 3D). Furthermore, TNFα treatment increased both exon 1a and 1b transcript levels (Figure 3E). Moreover, depletion of Fra-1 or c-Jun markedly impaired the TNFα-mediated induction of both transcripts (Figure 3E). Combined, these results indicate that TNFα regulates ZEB2 via two promoters dependent on AP-1 in TNBC cells.

### AP-1-mediated chromatin looping regulates ZEB2 transcription

**Figure 4:**
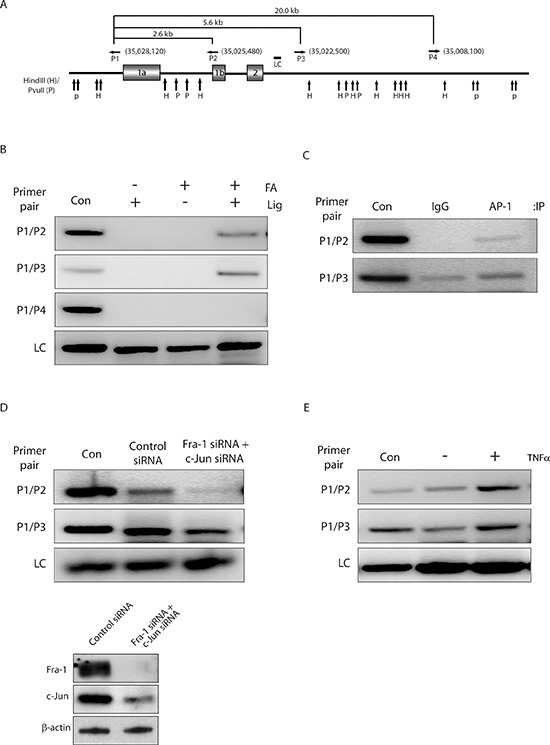
AP-1-mediated chromatin looping regulates ZEB2 transcription **(A)** Schematic of the *ZEB2* locus, showing 13833 kb region from base 34891668 to 35030000 (GenBank accession no. NW_004929304). Vertical arrows represent the locations of cleavage sites for HindIII (H) and PvuII (P). Horizontal arrows indicate locations and orientations of the combinations of primer sets used in 3C assay. Numbers in brackets indicate primer positions (numbering according to GenBank accession no. NW_004929304). Primer sequences were as follows: P1, GAGAGCATGATATTAAACGGCATGGGGTCTAAGTTTTGGA; P2, GGAGAATCCTGAAAACTCGGTGGAGGTTGGGAAGATGCTG; P3, TCTCTGAGTAATTTAGTAGACACTAAAATTACACCTAATG; P4, CGATCTTTGAATTTATCTGCAATGAGGTACAGCTTTAATG. The region amplified as a loading control (LC) is also indicated. **(B)** A looped topological conformation of the *ZEB2* promoter 1a and 1b is observed in BT549 cells. Results of 3C analysis with the indicated primer pairs on chromatin prepared from BT549 cells by crosslinking or not with formaldehyde (FA), and digestion with combination of HindIII and PvuII, followed by ligation (Lig) or not. PCR products from two primer pairs P1/P2 and P1/P3, which are closely located to exon 1a or exon 1b, were detected in samples from crosslinked chromatin with subsequent ligation (lane 4). As a negative control, primer pair P1/P4 generated no PCR product (panel 3). Lane Con shows positive control fragment, which indicates the expected size of PCR products in the 3C assay if the tested DNA ends are in close proximity. The sequences of primers for amplifying size marker templates will be provided on request. The loading control (LC) for each lane is shown in the bottom panel. **(C)** AP-1 associates with the juxtaposed promoter 1a and 1b. ChIP-loop analysis of putative gene loop region in chromatin prepared from BT549 cells using antibodies against IgG and AP-1 (a pool of Fra-1 and c-Jun antibodies). **(D)** Knockdown of AP-1 abolishes gene looping of promoter 1a and 1b. 3C assays performed on chromatin prepared from BT549 cells after transfection with control siRNA or siRNAs against Fra-1 and c-Jun for 72 hours. Bottom panels, Western blot analysis showing reduced protein levels of Fra-1 and c-Jun 72 hours after siRNA transfection in BT549 cells, as determined by using an anti-Fra-1 or anti-c-Jun antibody, respectively. β-actin was used as a loading control. **(E)** TNFα treatment increases gene looping. 3C assays performed on chromatin prepared from serum-starved BT549 cells treated with or without TNFα for 2 hours.

To investigate whether AP-1 binding to promoter 1b of the *ZEB2* gene also regulates the activity of promoter 1a by long range chromosomal interactions, we performed a 3C assay to determine whether there is a physical proximity between the *ZEB2* promoters 1a and 1b (Figure 4A). PCR bands resulting from the ligation products indicated that the promoter 1b was in close proximity to promoter 1a (Figure 4B). Negative control samples either from non-crosslinked chromatin with ligation by T4 ligase or from crosslinked chromatin without subsequent ligation did not generate any PCR bands. These results clearly demonstrate that promoters 1a and 1b of the *ZEB2* gene are in close proximity via chromatin looping. To further evaluate the role of AP-1 as a direct regulator of the loop formation between the two promoter regions, the ChIP-loop technique was used to determine whether AP-1 is physically associated with the gene loop. Our results demonstrated an association of AP-1 with the *ZEB2* gene loops (Figure 4C).

To address whether AP-1 is required for the gene looping within the *ZEB2* gene, we determined the effect of AP-1 depletion on the promoter 1a and 1b interaction. Western blot analysis confirmed that siRNA targeted to Fra-1 or c-Jun efficiently reduced the expression of Fra-1 or c-Jun (Figure 4D). 3C assays revealed that knockdown of Fra-1 or c-Jun impaired gene loop formation (Figure 4D), indicating that AP-1 is a critical mediator of long-distance interaction between the 1a and 1b promoters. Finally, treatment of cells with TNFα increased long-range interaction between the 1a and 1b promoters (Figure 4E), which correlated with TNFα-mediated induction in the mRNA levels of the exon 1a and 1b containing transcripts (Figure 3E).

Together, these results clearly demonstrated that AP-1, when activated by TNFα, binds to a site in the promoter 1b region of the *ZEB2* gene from which it regulates the expression of both promoter 1b and promoter 1a, the latter via mediating long-range chromatin interactions.

## DISCUSSION

It has become increasingly clear that inflammatory cytokines such as TNFα facilitate both tumor development and metastatic progression [25]. TNFα is a major inflammatory cytokine shown to be highly expressed in breast carcinomas [6]. TNFαexpression is also associated with tumor metastasis of breast cancer [26]. Our study provides several insights into the regulation of inflammatory cytokine-induced EMT that contributes to increases in invasive behavior of TNBC cells. We report that TNFα-induced EMT in TNBC cells functions via activation of AP-1 signaling, which in turn induces expression of the EMT regulator ZEB2. We also show that TNFα activates both the PI3K/Akt and MAPK/ERK pathways, which act upstream of AP-1. Moreover, we dissect mechanisms regulating expression of the *ZEB2* gene, whose expression is shown to be associated with AP-1-driven long-range combinatorial regulation.

Compared to other breast tumor subtypes, the PI3K/AKT pathway is enhanced in TNBC, and plays a critical role in promoting EMT and invasion in various cancers [27]. Furthermore, AP-1 components can be activated by MAPK/ERK signaling pathway [28]. In this study, we demonstrate that these two signaling pathways are upstream regulators of AP-1 in response to TNFα. We find that LY294002 and PD98059 treatment led to a significant down-regulation of Fra1and c-Jun proteins, suggesting that the activation of the PI3K/Akt and MAPK/ERK pathways is responsible for TNFα-mediated AP-1 up-regulation in TNBC cells.

Our study reveals a critical role for AP-1–ZEB2 axis in TNFα-initiated EMT. Although previous research has implicated the interplay between inflammatory cytokines and the activation of different signaling pathways, like Wnt, Notch and NF-κB signaling in the regulation of EMT-like processes [29], our results further suggest that AP-1 signaling is necessary for TNFα-induced EMT. The inflammatory mediators, such as TNFα, TGFβ and IL-6, in regulation of Snail have been implicated in the initiation of EMT [30–32]. It has been demonstrated that Snail enhances TNFα-induced cancer cell migration and invasion in breast cancer cells, through stabilization of NF-κB activation. [32]. However, much less is known about the regulation of the other regulators. Consistent with our data on the role of ZEB2 in TNFα-induced EMT, Vandewalle et al. [33] showed that exogenous ZEB2 expression in a colon cancer cell line induced a dramatic morphological conversion, from an epithelial cell state to a fibroblast-like phenotype. Expanding on our previous findings that ZEB2 caused an invasive phenotype in TNBC cells [13], this study indicates that ZEB2 induction mediated by the inflammatory cytokine TNFα is critical for EMT and thus provides a plausible molecular mechanism contributing to TNBC aggressiveness.

The human *ZEB2* gene includes two promoters followed by distinct non-coding first exons, which are located 2.2 kb apart and are spliced to the common exon 2 containing the translation initiation site. This study analyzed for the first time the distribution of the two transcripts of ZEB2 directed from distinct promoters in a number of primary breast tumors as well as a number of cell lines. Both transcripts were highly expressed in TNBC cells such as MDA-MB-231, BT549, Hs578T and MDA-MB-157, representing the invasive breast cancer subtype. This result is in agreement with our previous findings that high levels of a total pool of ZEB2 mRNAs were detected only in TNBC cell lines [13]. In relevance to these data, it has been reported that ZEB2 expression is higher in various cancers compared with normal tissues [34, 35]. We showed that transcripts from both promoters were expressed in breast cancer tissues, indicating that both promoters are targets for regulation of ZEB2 expression in breast cancer. Interestingly, we found that the exon 1a transcript was detected in all analyzed breast tumors samples, whilst exon 1b transcript displayed a more restricted expression. This may be associated with distinct breast cancer subtypes included in the study. Many genes are known to be expressed with alternative 5′UTRs often originating from distinct promoters. 5′UTR regions can influence mRNA stability and translational efficiency and thus differential expression of transcripts with different 5′UTRs is a means to modulate protein expression [36]. The potential significance of the expression of alternative UTRs is related to tissue-specific expression patterns and a contribution of dysregulated expression of alternative UTRs to the development of disease [36]. The role of the two ZEB2 promoters on human tissue-specific expression patterns and ZEB2 protein expression levels needs to be further investigated.

It is increasingly recognized that the expression of multiple genes may be coordinately regulated through chromatin looping [37]. In this study, we observed that AP-1 could regulate expression of two distinct ZEB2 mRNAs, which differ in their 5′UTR but translate the same protein, through the use of alternative promoters by *in vivo* long-range chromosomal interactions. However, only one transcript variant has an AP-1 binding site in its promoter region, raising the important question of whether binding of AP-1 to the AP-1 element located in the proximal promoter may act long-range to activate distal promoter through DNA looping. We used the 3C assay to demonstrate the existence of AP-1-dependent *in vivo* long-range chromosomal interactions between ZEB2 distal and proximal promoters. Moreover, knockdown of AP-1 abolished chromatin loop formation. Thus, our data provide a novel mechanism of AP-1-driven long-range combinatorial regulation. Such chromatin-based transcription mechanisms have been reported for several genes. For example, Gong et al. [38] have shown that BCL2 gene expression is controlled by chromatin looping between a 3′-UTR region and BCL2 promoter.

We present evidence for chromatin looping between the *ZEB2* promoters 1a and 1b (located ~2.4 kb apart). DNA looping over ~100 bp to 10 kb distances that bring regulatory elements into the proximity of promoters, contributing to gene activation, has been documented for many genes, including the CD68 gene spanning over a 2.5 kb distance [39], the HO-1 gene over a 6.0 kb distance [40], and the insulin gene spanning a region of 1.4 kb [41]. More recently, comprehensive chromatin interactions were mapped in human cells using a genome-wide 3C analysis method (Hi-C). It was demonstrated that the sizes of the identified interacting DNA loci range from several hundred bp to over 50 kb, with a median of 10.5 kb [14], further emphasizing the occurrence of looping interactions over variable distances.

In the present work, we demonstrate that the pro-inflammatory cytokine TNFα-triggered EMT of TNBC cells is mediated through AP-1-induced ZEB2 up-regulation. We dissect mechanisms underlying regulation of the *ZEB2* gene, whose expression is shown to be associated with AP-1-driven long-range combinatorial regulation. Our study not only reveals a critical mechanism underlying inflammation-induced metastatic potential in TNBC but also provides evidence of AP-1 and its upstream ERK and Akt signaling, and ZEB2 as potential drug targets for reversing EMT, the key event in the acquisition of invasive potential.

## METHODS

### Cell lines and reagents

T47D, MCF-7, MDA-MB-175, ZR-75–1, SK-BR-3, HCC1569, MDA-MB-453, MDA-MB-231, BT549, Hs578T, and MDA-MB-157 cells were obtained from American Type Culture Collection. Human recombinant TNFα was purchased from Roche, and LY294002 and PD98059 were from Sigma.

### Reverse transcription-PCR

Reverse transcription-PCR (RT-PCR) was done using standard procedures. The primers used in each reaction were as follows: Exon 1a ZEB2 transcript, forward 5′-TTCTCACCATTTCTGGCCAAA-3′ and reverse 5′-CACCCTCCCTTATTTCATCTTCCT-3′; Exon 1b ZEB2 transcript, forward 5′-GGGTGCAACACACCAAACAA-3′ and reverse 5′-CACCCTCCCTTATTTCATCTTCCT-3′; β-actin, forward 5′-CAAGGCCAACCGCGAGAAGAT-3′ and reverse 5′-GTCCCGGCCAGCCAGGTCCAG.

### Human breast tumor samples

The 16 breast tumor samples in this study have been previously described [42]. The studies were approved by the ethical committee of the Karolinska Institutet.

### Chromatin immunoprecipitation

BT549 cells were cultured on 150 mm dishes and were starved in RPMI 1640 medium containing 1% fetal bovine serum (FBS) overnight and then treated with or without TNFα (10 ng/ml) for 2 hours. Chromatin immunoprecipitation (ChIP) was performed as previously described [42]. The following antibodies were used: anti-p-Fra-1 (Ser265; #3880) from Cell Signaling Technology; anti-Fra-1 (R-20), anti-c-Jun (H-79) and normal rabbit IgG from Santa Cruz Biotechnology. The immunoprecipitated DNA was amplified by qPCR using primers to a known AP-1 binding region in the ZEB2 promoter 1b. Primer sequences used were: forward 5′-TGACCGTATGAGGGAATGCA-3′ and reverse 5′-CCG CCTAGCCCTGAGTCAA-3′

### Western blotting

Western blot analyses were performed as previously described [42]. Antibodies used were anti-Fra-1 (R-20; sc-605), anti-c-Jun (H-79; sc-1694), anti-p-c-Jun (KM-1; sc-822) and anti-ZEB2 (E-11; sc-271984) from Santa Cruz Biotechnology; anti-E-cadherin (610182), anti-N-cadherin (610920), and anti-fibronectin (610077) from BD Biosciences; anti-β-actin from Sigma; anti-p-Fra-1 (Ser265; #3880), anti-Akt #9272, anti-phospho-Akt (Ser473; #9271), anti-ERK, and anti-phospho-ERK were from Cell Signaling Technology.

### qPCR

qPCR was performed in a 7500 Fast Real-Time PCR (Applied Biosystems, Foster City, CA) with Fast SYBRGreen Master mix (Applied Biosystems) according to conditions specified by the manufacturer. The specificity of all primer pairs was checked with melting curve analysis. Primer sequences will be provided on request.

### RNA interference

BT549 cells were cultured to 50% confluency and transfected with Fra-1 ON-TARGET plus SMARTpool (Thermo Scientific; L-004341–00-0010), c-Jun ON-TARGET plus SMARTpool (Thermo Scientific; L-003268–00-0010), ZEB2 ON-TARGET plus SMARTpool (Thermo Scientific; L-006914–02-0010) or nontargeting pool (Thermo Scientific; D-001810–10-20) using the INTERFERin siRNA transfection reagent (Polyplus) according to the manufacturer's instructions.

### 3C

3C assays were performed in BT549 cells as described previously [43]. Briefly, BT549 cells were formaldehyde cross-linked at room temperature for 10 min. After the addition of glycine for 5 min, cells were pelleted and intact nuclei were resuspended in lysis buffer (Sigma-Aldrich Sweden AB) containing protease inhibitors (Roche Diagnostics GmbH, Mannheim, Germany). Appropriate amounts of the chromatin solutions were subjected to overnight digestion with *Hin*dIII and *Pvu*II (New England Biolabs, Hitchin, UK). After ligation by T4 ligase, crosslinking was reversed and DNA extracted by phenol/chloroform before precipitation by ethanol. ChIP-loop was carried out as described [43]. The crosslinked chromatin fragments were immunoprecipitated with IgG or a pool of anti-Fra-1 and anti-c-Jun antibodies.

## Abbreviations

TNBC: triple-negative breast cancer
EMT: epithelial-mesenchymal transition
ER: estrogen receptor
3C: chromosome conformation capture
ChIP: Chromatin immunoprecipitation
